# Effect of systolic blood pressure fluctuations during resuscitation on postoperative complications following meningioma surgery: A retrospective observation study

**DOI:** 10.1097/MD.0000000000032259

**Published:** 2022-12-09

**Authors:** Dong Xue Luo, Zi Chuan Yue, Min Shi, Xing Jie Guo, Ya Qing Zhou, Lu Yi Shao, Miao Miao Xu, Jie Jie Zhou, Li Xiang Yu, Manlin Duan

**Affiliations:** a Department of Anesthesiology, Affiliated Jinling Hospital, Medical School Nanjing University, Nanjing, China; b Department of Anesthesiology, BenQ Medical Center, The Affiliated BenQ Hospital of Nanjing Medical University, Nanjing, China; c College of Anesthesiology, Xuzhou Medical University, Xuzhou, China.

**Keywords:** anesthesia resuscitation, meningioma surgery, postoperative complications, postoperative length of stay, systolic blood pressure fluctuations

## Abstract

It is unclear whether blood pressure variability in the post-anesthesia care unit is associated with postoperative complications. This study aims to characterize the impact of blood pressure fluctuations on postoperative complications and postoperative length of stay after meningioma surgery. Adult meningioma patients undergoing general anesthesia were retrospectively recruited. The principal exposure was blood pressure variability in the post-anesthesia care unit, calculated by noninvasive blood pressure measurements. The primary outcome was major postoperative complications, defined as II or higher in the Clavien-Dindo classification grades. Secondary outcomes included healthcare resource utilization parameters among patients. Multivariable logistic regression was used and adjusted for potential confounding variables. Data sensitivity analyses were performed via different variable transformations and propensity score matching analyses. A total of 578 patients qualified for the study, and 161 (27.9%) cases experienced postoperative complications. The multivariable analysis found that increased systolic blood pressure variability in the post-anesthesia care unit was associated with postoperative complications (adjusted odds ratio [aOR] = 1.15; 95% confidence interval [CI], 1.09–1.22, *P* < .001) and prolonged postoperative length of stay (adjusted regression coefficients [β] = 1.86; 95% CI, 0.58–3.13, *P = *.004). Patients with postoperative complications had a higher frequency of intensive care admission (44.1% vs 15.3%), major postoperative interventions (6.6% vs 0%), and 30-day readmission (5.0% vs 0.7%). Systolic blood pressure fluctuations during resuscitation have an independent impact on postoperative complications and postoperative length of stay following meningioma surgery.

## 1. Introduction

Meningiomas are the most common primary intracranial tumor, comprising 36.6% of all primary central nervous system tumors in the USA.^[1]^ Although meningiomas are highly prevalent as a primary benign disease, surgical resection remains a critical treatment paradigm. Evidence shows that, like other neurosurgical procedures, meningiomas are frequently associated with poor prognoses such as focal neurological deficits, seizures, and decreased quality of life.^[2]^ Several studies have explored the risk factors related to postoperative complications (POCs) in meningiomas, with varying incidence estimates among different surgical populations (15–35.8%).^[3–5]^ POCs of meningiomas after general anesthesia deserves greater attention because of their association with more complex postoperative medical care, prolonged postoperative length of stay, long-term disability, and mortality of patients.^[3]^

As demonstrated in previous literature, abnormal blood pressure changes lead to varying levels of target organ damage.^[6]^ The prognosis of surgical procedures is significantly correlated to the management of perioperative anesthesia and the degree of circulatory stability. Liu et al^[7]^ demonstrated that one of the leading causes of rebleeding after a craniotomy was the increased incidence of postoperative blood pressure fluctuations. In prior studies, blood pressure fluctuations were often assessed based on mean values or a specific threshold range without considering the intrinsic dynamics.^[8–11]^

Evidence shows that persistent, significant intraoperative blood pressure fluctuation affects the outcome of critically ill and surgical patients.^[12,13]^ However, little attention has been paid to the blood pressure changes in the post-anesthesia care unit (PACU) after general anesthesia. The relationship between postoperative blood pressure fluctuations and postoperative outcomes for patients after meningioma resection has not been reported previously. Therefore, we conducted a retrospective cohort study to determine whether increased blood pressure fluctuations in the PACU demonstrate increased risks of POCs and prolonged postoperative length of stay (PLOS) following meningioma surgery.

## 2. Methods

### 2.1. Study design and eligibility criteria

Meningioma resection was determined according to the international ICD-9 code and a retrospective cohort study was conducted on adult meningioma surgery patients following general anesthesia between January 2019 and June 2020. The study was approved on January 21, 2022 by the Ethics Committee of the Jinling Hospital, affiliated with the Medical School of Nanjing University (no. 2022NZKY-010-01), with a waiver of informed consent. The study was conducted in accordance with the principles set forth in the Helsinki Declaration. The primary outcomes and sensitivity analyses were defined and established before the initiation of the study design. We followed the Strengthening the Reporting of Observational Studies in Epidemiology guidelines. Patients aged 18 years or older at the time of inclusion and who had been diagnosed with meningioma were eligible for this study. The exclusion criteria included no admission to the PACU for recovery, incomplete blood pressure data in the PACU, other intracranial lesion resections during the meningioma procedure, or a pathological diagnosis of the meningioma as a different tumor type. The missingness of data was assumed to be at random.

### 2.2. Exposures and data collection

The principal exposure, blood pressure variability in the PACU, was defined as the magnitude of the coefficient of variation (CV). Blood pressure variability was calculated using the CV = standard deviations/mean formula.^[14]^ After the patient’s admission to the PACU, arterial blood pressure was measured continuously at 5-minute intervals using an oscillometric upper-arm cuffs and recorded. Two researchers performed a rigorous screening and exclusion process for blood pressure data during resuscitation, including the removal of obviously erroneous blood pressure values and a final consistency test, which preserved the true blood pressure values of the patients. Data pertaining to demographic characteristics were obtained from the Jinling Hospital institution’s Electronic Medical Records. Recorded variables included sex, age, comorbidities, World Health Organization (WHO) tumor grade, and other tumor characteristics. Perioperative data included the date and duration of surgery, fluid management strategies, intraoperative blood transfusions, intraoperative hypertension, whether the patient received tracheal extubation in the PACU, and resuscitation time (defined as the time from when the patient entered to exited the PACU). These variables were all retrieved from the Surgical Anesthesia Information Records. Specific postoperative complications were summarized with reference to the authoritative literature and based on the international ICD-9 code.^[15]^ We collected specific postoperative complications from the Electronic Medical Records, including intracerebral hemorrhage and edema, intracranial infection, cerebrospinal fluid leakage, pulmonary infections, etc.

### 2.3. Outcomes

The primary outcome was the incidence of major POCs, which are defined as any deviation from the ordinary postoperative course requiring pharmacological treatment or interventional procedures, rated as II or higher in the Clavien-Dindo classification (CDC) grades.^[16]^ Secondary outcomes included following healthcare resource utilization (HCRU) parameters, such as duration of Neurosurgical Intensive Care Unit, needs for major postoperative interventions, and 30-day readmission. We defined 30-day readmission as all-cause hospitalizations within 30 days of discharge and were limited to readmission to our hospital.

### 2.4. Sensitivity analyses

Several sensitivity analyses were performed, including different variable classifications and definitions, the order in which variables are entered into the model, and subgroup analyses and interactions between variables. Additional adjustments for confounding variates were used to minimize bias, such as propensity score matching (PSM). We performed a one-to-one matching without replacement using the nearest-neighbor matching algorithm with caliper widths equal to 0.1. The matching effect of PSM was measured by the covariate-balanced plot.

### 2.5. Statistical analysis

Since no prior data were available on the primary outcome measure, we performed a post hoc power calculation. Assuming the incidence of in-hospital POCs of 15.5% of patients with lower systolic pressure variability (SBPV), as seen in the first-year data, increased SBPV increases the risk of in-hospital POCs by an absolute 8%. The study would provide a power of 0.80 to detect this difference at a two-sided significance level of 0.05. Considering an exclusion rate of 5%, the current required sample size was estimated at 536 patients.

Continuous variables were summarized as means (standard deviation) or medians (interquartile ranges) and categorical variables as frequencies and proportions. Student *t* tests and Mann–Whitney *U* tests were used to assess the differences in numerical data between groups, and the χ^2^ tests for comparisons between categorical variables.

The relationship between blood pressure variability and in-hospital POCs was assessed using adjusted odds ratios (aORs) with 95% confidence intervals (CIs). For all analyses, we required a minimum of 10 events per predictor to avoid overfit models.^[16]^ Potentially risk factors were adopted and adjusted from preliminary evidence that might affect in-hospital POCs.^[2–5,17]^ Subgroup analysis of in-hospital POCs stratifying the cohort was conducted as a post hoc study. The effect of blood pressure variability on PLOS was summarized using a univariable and multivariable generalized linear model with an adjusted β valve and 95% CIs. The fitted model used a gamma distribution and fully accounted for variable multicollinearity. Restricted cubic splines were applied to delineate the curve of associations. Secondary outcomes were considered exploratory.

A series of sensitivity analyses were performed. Multivariable logistic regression models were used to evaluate associations between all confounding variables with and without SBPV and in-hospital POCs. The fitted model discrimination was described by the area under receiver operating characteristics curves. The SBPV values were a natural log transformed to reduce the influence of extreme values, as both continuous variables and quartiles. A PSM cohort was carried out at a ratio of 1:1 by logistic regression to reduce the effects of confounders and selection bias.

All analyses were performed using IBM SPSS Statistics for Windows, Version 26.0 (IBM Corp., Armonk, NY). A two-sided *P* value < 0.05 was considered statistically significant.

## 3. Results

### 3.1. Study cohort and characteristics

**Figure 1.**
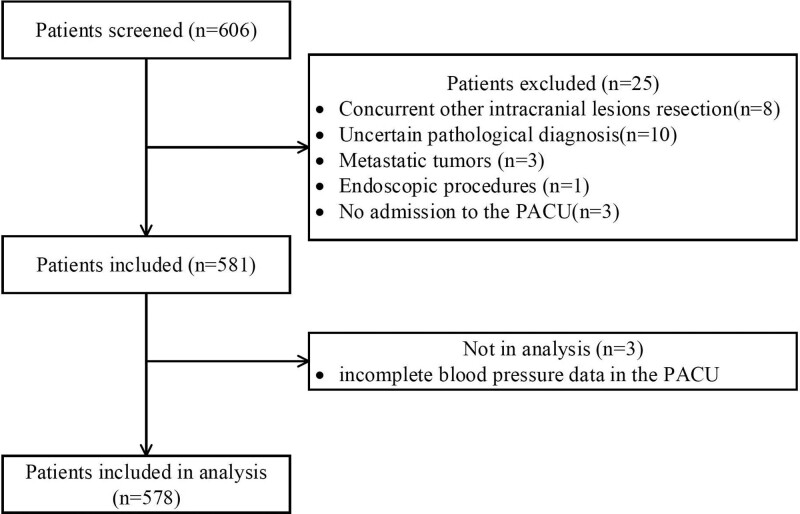
Flow chart of patient data. PACU = post-anesthesia care unit.

A total of 606 patients with meningioma were initially included, and 3 patients who were not admitted to PACU for recovery, 8 patients who underwent concurrent resection of other intracranial lesions, 13 patients with pathological diagnosis of other tumors, 1 endoscopic procedure, and 3 patients with incomplete blood pressure data in PACU were excluded. Finally, 578 patients were included for analysis (Fig. 1). The baseline and perioperative characteristics of patients are presented in Table 1. The mean (SD) age at meningioma surgery was 55.2 (0.5) years, and 445 (77.0%) were female. The median of PLOS for the entire cohort was 10 days. Postoperative complications were presented in 161 (27.9%) patients. Demographic characteristics, surgical and anesthetic data, postoperative events, and postoperative complications are described in detail in Tables S1–S4, Supplemental Digital Content (http://links.lww.com/MD/I113).

**Table 1:** Patients’ characteristics.

	POCs group	Non-POCs group	*P* value
No. of patients	161	417	
Patient characteristics			
Age, yr, mean (SD)	56.7 (12.2)	54.6 (10.8)	.044
Female, sex, n (%)	121 (75.2)	324 (77.7)	.515
Preoperative characteristics			
Comorbidities, n (%)			
Hypertension	60 (37.3)	114 (27.4)	.021
Diabetes mellitus	20 (12.4)	42 (10.1)	.558
Neurological diseases	36 (22.4)	51 (12.3)	.002
Cardiovascular disease	9 (5.6)	15 (3.6)	.284
Neurosurgical history, n (%)	18 (11.2)	32 (7.7)	.182
Charlson comorbidity index, M (IQR)	4 (1)	3 (1)	.128
WHO tumor grade, n (%)			
Ⅰ	139 (86.3)	379 (91.1)	.043
Ⅱ	17 (10.6)	33 (7.9)	
Ⅲ	5 (3.1)	4 (1.0)	
Maximum tumor diameter, mm, M (IQR)*	39.2 (16.6)	32.2 (15.0)	<.001
Recurrent tumors, n (%)	17 (10.6)	32 (7.7)	.268
Multiple tumors, n (%)	12 (7.5)	10 (2.4)	.004
Preoperative anemia, n (%)	11 (6.8)	26 (6.2)	.793
ASA classification, n (%)			
Ⅱ	141 (87.6)	387 (93.0)	.051
Ⅲ	20 (12.4)	28 (6.7)	
Ⅳ	0 (0.0)	1 (0.2)	
Perioperative characteristics			
Operation time, min, mean (SD)	303.8 (141.0)	230.3 (91.4)	<.001
Total liquid, mL, M (IQR)	2000.0 (1000)	1500.0 (500)	<.001
Total blood loss, mL, M (IQR)	400.0 (300)	300.0 (200)	<.001
Blood transfusion, n (%)	33 (20.5)	47 (11.3)	.004
Intraoperative hypertension, n (%)	47 (29.2)	94 (22.5)	.095
Intraoperative hypotension (MAP < 65 mm Hg), n (%)	12 (7.5)	30 (7.2)	.576
Tracheal unextubation, n (%)	23 (14.3)	14 (3.4)	<.001
SBPV%, mean (SD)	8.1 (3.4)	6.2 (3.1)	<.001
DBPV%, mean (SD)	7.3 (2.8)	7.1(3.0)	.418
Postoperative events			
PLOS, M (IQR)	15 (13)	9 (4)	<.001
30 day readmission, n (%)	8 (5.0)	3 (0.7)	.003
90 day readmission, n (%)	13 (8.1)	7(17)	<.001

Data were presented as frequency (prevalence in %) or means (SD), or medians (IQR).

ASA = American Society of Anesthesiologists Physical Status Classification, CDC = Clavien-Dindo Classification, DBPV = diastolic blood pressure variability, IQR = interquartile range, LOS = length of stay, M = Median, PLOS = postoperative length of stay, POCs = postoperative complications, SBPV = systolic blood pressure variability, SD = Standard deviations., WHO = World Health Organization.

* Missing data.

### 3.2. Association between systolic blood pressure fluctuations and POCs

**Figure 2.**
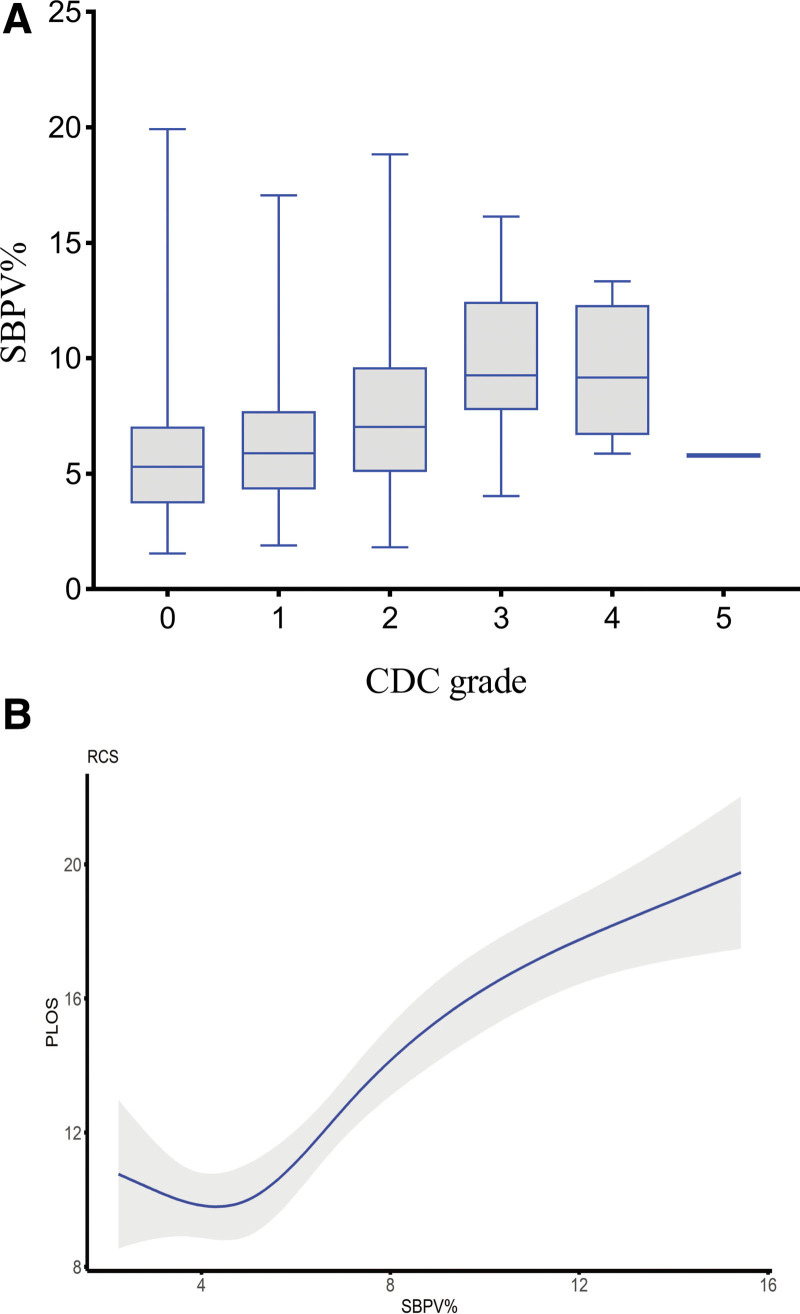
(A) Association of increased systolic blood pressure variability with CDC grades. (B) The restricted cubic spline of the association between increased systolic blood pressure variability and postoperative length of stay. CDC = Clavien-Dindo classification, PLOS = postoperative length of stay, SBPV = systolic blood pressure variability.

An analysis of perioperative factors predicting POCs were preoperative comorbidities, WHO classification Ⅲ, operation time ≥ 3 hours, more intraoperative fluid administration, and tracheal unextubation in the PACU (Table 2). Increased SBPV in the PACU was also associated with POCs (aOR = 1.15; 95% CI, 1.09–1.22, *P* < .001) when added to the regression model. For the sensitivity analysis, the inclusion of SBPV in the regression model did not qualitatively change estimates of other covariates; however, improved the model’s prediction accuracy (Table 2). Meanwhile, ln (SBPV) was robustly linked with POCs (aOR = 3.39; 95% CI, 2.16–5.13, *P* < .001), and the results were also similar when analyzed as quartile variables (Table S5, Supplemental Digital Content, http://links.lww.com/MD/I114). The association between increased SBPV and CDC grades was presented in Figure 2A. A subgroup analysis of POCs described the effect of SBPV in each stratum compared to no adjustment, and no interactions were detected (Figure S1, Supplemental Digital Content, http://links.lww.com/MD/I117).

**Table 2:** Adjusted association of perioperative characteristics and systolic blood pressure variability with postoperative complications.

Risk factors	Adjusted odds ratio (95% CI) for POCs			
	Perioperative characteristics alone*		Perioperative characteristics and SBPV†	
	aOR (95% CI)	*P* value	aOR (95% CI)	*P* value
Comorbidities	1.83 (1.22–2.73)	.003	1.62 (1.07–2.44)	.022
WHO classification	5.16‡ (1.28–16.01)	.015	3.19‡ (2.10–20.85)	.030
Operation time ≥ 3 h	1.92 (1.13–3.28)	.016	1.76 (1.03–3.02)	.039
Total liquid	1.00 (1.00–1.01)	<.001	1.00 (1.00–1.01)	<.001
Tracheal unextubation	3.30 (1.45–6.34)	.003	3.08 (1.45–6.54)	.004
SBPV	NA	NA	1.15 (1.09–1.22)	<.001

Statistical analyses were performed using multivariable logistic regression with no POCs as the reference group. aORs are reported for logistic regression analyses with 95% CIs. A *P* value of < .05 was statistically significant.

95% CI = 95% confidence interval, aOR = adjusted odds ratio, NA = not available, PACU = post-anesthesia care unit, POCs = postoperative complications, SBPV = systolic blood pressure variability, WHO = World Health Organization.

* c-index for the fitted regression model is 0.699.

† c-index for the fitted regression model is 0.738.

‡ WHO Ⅲ aORs, using WHO Ⅰ as the reference.

### 3.3. Association between systolic blood pressure fluctuations and PLOS

In the adjusted generalized linear model, the association between increased SBPV in the PACU and prolonged PLOS was statistically significant (adjusted β = 1.86; 95% CI, 0.58–3.13, *P = *.004), and the results were similar in sensitivity analyses (Table S6, Supplemental Digital Content, http://links.lww.com/MD/I115). The restricted cubic spline described a monotonic increase in PLOS with increased SBPV values, as shown in Figure 2B.

### 3.4. Secondary outcomes

Table 3 contains the HCRU results among patients in the entire cohort. Compared to the population without POCs, patients with postoperative complications had higher rates of intensive care admissions and readmissions, in addition to increased postoperative medical needs such as secondary surgery reinterventions, postoperative blood transfusions, tracheotomies, and ventilator-assisted breathing.

**Table 3:** Healthcare resource utilization in the entire cohort.

	CDC grade < 2	CDC grade ≥ 2	*P* value
	(n = 417)	(n = 161)	
Major interventions, n (%)			
Secondary surgery	0 (0)	14 (8.7)	<.001
Postoperative blood transfusion	0 (0)	16 (9.9)	<.001
Tracheotomy and ventilator-assisted breathing	0 (0)	16 (9.9)	<.001
NICU LOS, M [25th, 75th]	1 [1, 1]	1 [1, 3]	<.001
PLOS, M [25th, 75th]	9 [8, 12]	15 [9, 22]	<.001
30 day readmission, n (%)	3 (0.7%)	8 (5.0%)	.003
90-day LOS, M [25th, 75th]	0 [0, 0]	0 [0, 0]	<.001
Total hospitalization costs, yuan, mean (SD)	73700 (766.6)	100294 (2992.2)	<.001

Data were presented as frequency (prevalence in %) or means (SD), or medians (IQR, 25th–75th percentile). A *P* value of < .05 was statistically significant.

CDC = Clavien-Dindo Classification, IQR = interquartile range, LOS = length of stay, M = Median, NICU = Neurosurgical Intensive Care Unit, POCs = postoperative complications, SD = standard deviations.

### 3.5. PSM sensitivity analyses

Following propensity score matching, 212 patients were incorporated into the final analysis. The significant associations between systolic blood pressure fluctuations and the postoperative outcomes were validated in both the PSM and the entire cohort (Table 4). Baseline and perioperative covariates of patients and the matching effect of each covariate before and after PSM were shown in the supplementary material (Table S7, Supplemental Digital Content, http://links.lww.com/MD/I116 and Figure S2, Supplemental Digital Content, http://links.lww.com/MD/I118). However, the 95% CI limits for these adjusted associations were maybe less precise in the reduced sample size.

**Table 4:** Association of systolic blood pressure variability with the postoperative outcome in propensity score matching cohort and entire cohort.

	Postoperative outcome	OR (95% CI)	*P* value
PSM cohort (n = 342)			
Unadjusted	In-hospital POCs	1.16 (1.05–1.29)	.008
Adjusted	In-hospital POCs	1.14 (1.03–1.27)	.014
Unadjusted	PLOS	2.15 (0.19–4.09)	.032
Adjusted	PLOS	1.85 (0.10–3.57)	.038
Entire cohort (n = 578)			
Unadjusted	In-hospital POCs	1.18 (1.12–1.25)	<.001
Adjusted	In-hospital POCs	1.15 (1.09–1.22)	<.001
Unadjusted	PLOS	0.87 (0.67–1.07)	<.001
Adjusted	PLOS	1.86 (0.58–3.13)	.004

Statistical analyses were performed using multivariable logistic regression with no postoperative complications as the reference group. Results are reported as OR or adjusted OR with 95% CIs for the primary exposure variable, showing results for patients experiencing in-hospital POCs. A *P* value of < .05 was statistically significant.

95% CI = 95% confidence interval, CDC = Clavien-Dindo Classification, OR = odds ratio, PLOS = postoperative length of stay, POCs = postoperative complications, PSM = propensity score matching, SBPV = systolic blood pressure variability.

## 4. Discussion

Our findings reveal that among patients having meningioma surgery, increased systolic blood pressure fluctuations during resuscitation were significantly associated with increased risks of postoperative complications and prolonged postoperative length of stay. Based on the CDC grades, the incidence of POCs was 27.9%, and is comparable to the previous study by Zhao et al in 2018.^[5]^ The robustness of the results was further demonstrated by a series of sensitivity analyses.

The Clavien-Dindo classification is a common method used to classify complications.^[18]^ To avoid ambiguity, we defined severities of postoperative complications rated as II or higher by the CDC grades.^[19]^ In a prospective study, Woodfield et al^[20]^ found that approximately one-third of complications occur between hospital discharge and 30 days postoperatively. Since only in-hospital POCs were considered, as reported in other studies,^[21,22]^ our results might have underestimated the incidence of POCs. In addition to the hemodynamic variability observed in the PACU, our risk factors for POCs after meningioma surgery were largely congruent with those previously reported in the literature: preoperative comorbidities, higher WHO classification, longer operation time, and more intraoperative fluid administration.^[3–5,17]^ The consistency of our findings further supports the validity of the multivariable regression models.

We selected blood pressure as the clinical biomarker due to its ease of measurement and clinical applicability. Our variability calculation included increases and decreases in blood pressure, which were not comprehensively considered in previous studies. In two randomized studies,^[8,9]^ blood pressure fluctuations were evaluated as changes beyond a specific threshold range. In the study by Liu et al^[10]^ blood pressure fluctuations were considered the absolute difference between two consecutive measurements. Furthermore, a 2019 prospective cohort study defined blood pressure fluctuation as the difference between the highest and lowest mean arterial pressure (MAP).^[11]^ Blood pressure assessments focusing on a single value may introduce potential bias. The parameter CV used in our data differs from a recent study by Hirsch et al,^[23]^ which quantified blood pressure fluctuations during surgery as the variance of blood pressure. Our data incorporated the blood pressure means into the calculation and considered the variance. Therefore, it may be more scientific to include blood pressure variability as a risk predictor rather than a threshold for change or an absolute difference.

Our current findings and previous studies^[12,14,24,25]^ showed a relationship between persistent blood pressure fluctuations and adverse outcomes, including increased mortality. However, some observations were not limited to brain surgeries. Our data showed an apparent postoperative fluctuation of systolic blood pressure among patients with POCs following meningioma surgery. Some studies have reported that blood pressure fluctuations were related to cerebral hemorrhage after a craniectomy or enlargements of the hemorrhage hematoma.^[26]^ Early stabilization of blood pressure could significantly inhibit hematoma enlargement and improve the prognosis of these patients. Furthermore, prior studies have confirmed dramatic fluctuations in MAP as an independent predictor of poor neurological prognosis.^[27–29]^ The exact role of blood pressure fluctuations is less well described. In 2014, two studies both showed that higher magnitudes of MAP above the upper threshold of cerebral autoregulation are associated with a higher risk of complications.^[30,31]^ They proposed that blood pressure variability above the vasomotor constraints of autoregulation might lead to endothelial damage and a compromised blood-brain barrier. In addition, ischemia-reperfusion injury caused by sustained blood pressure fluctuations may also contribute to the development of POCs.^[32]^ Accordingly, optimizing the magnitude of blood pressure fluctuations during resuscitation to remain within the cerebrovascular autoregulation range might provide a possible strategy for modifying the risk for POCs and reducing PLOS.

Some possible reasons for blood pressure fluctuations were summarized as:^[33]^ Patient-related factors: history of hypertension, advanced age, comorbid endocrine disorders, etc.; Anesthetic-related factors: chills, postoperative pain, hypoxia and CO2 accumulation, inadequate depth of sedation; and Medical intervention-related factors: suctioning operations, extubation stimulation, etc. In our cohort, both postoperative systolic and diastolic blood pressure variabilities were evaluated. In a 2019 retrospective study, Liu et al^[10]^ noted that greater postoperative fluctuation of systolic blood pressure was related to increased neurological and overall complications among glioblastoma patients. Our results affirmed the role of increased systolic blood pressure fluctuation rather than diastolic blood pressure for POCs and prolonged PLOS, which is consistent with Liu’s and Muntner’s findings.^[10,34]^ These might suggest that fluctuations of systolic blood pressure in the PACU deserve more attention. Moreover, the underlying mechanisms as well as the causality of this relationship remain to be elucidated at the blood pressure levels observed in the present study.

The current study also provides evidence of HCRU. During the postoperative period, patients who developed POCs were admitted to the Neurosurgical Intensive Care Unit more frequently. Several studies highlighted medical resource care for POCs after meningioma surgery.^[35–37]^ Consistent with previous studies,^[36]^ we found that increased systolic blood pressure fluctuations prolonged postoperative length of stays, increasing medical care in such patient populations. In contrast, the postoperative event data in our cohort was considered more comprehensively including hospital readmissions and complicated postoperative medical needs, such as the need for secondary surgery reinterventions, postoperative blood transfusions, and tracheotomies. Thus, our findings are complementary to prior studies.

There are several limitations to this study. Firstly, as this was a retrospective review, stratification analysis of the severity and duration of blood pressure in the PACU was not performed. Secondly, we could not provide causation evidence about those associations in this observational study. Meanwhile, the external validity of the results needs to be confirmed in different patient populations. Thirdly, this study included a heterogeneous population regarding different baseline and perioperative characteristics, for which PSM and a series of sensitivity analyses could not offset all biases.

In summary, under the actual data from meningioma surgery, we present evidence that increased systolic blood pressure fluctuations during resuscitation have an independent and significant impact on POCs and prolonged PLOS. Further studies are warranted to identify possible causal mechanisms underlying this association.

## Acknowledgments

The authors thank AiMi Academic Services (www.aimieditor.com) for English language editing and review services.

## Author contributions

**Conceptualization:** Dong Xue Luo, Manlin Duan.

**Data curation:** Zi Chuan Yue.

**Formal analysis:** Dong Xue Luo, Zi Chuan Yue, Jie Jie Zhou.

**Funding acquisition:** Manlin Duan.

**Investigation:** Dong Xue Luo, Min Shi.

**Methodology:** Dong Xue Luo.

**Resources:** Manlin Duan.

**Software:** Dong Xue Luo, Xing Jie Guo, Ya Qing Zhou, Jie Jie Zhou, Li Xiang Yu.

**Supervision:** Lu Yi Shao, Miao Miao Xu, Manlin Duan.

**Validation:** Ya Qing Zhou, Miao Miao Xu, Li Xiang Yu.

**Visualization:** Min Shi, Xing Jie Guo.

**Writing – original draft:** Dong Xue Luo, Zi Chuan Yue.

**Writing – review & editing:** Lu Yi Shao, Manlin Duan.

## Supplementary Material



## References

[1] Ostrom QT Gittleman H Xu J. CBTRUS statistical report: primary brain and other central nervous system tumors diagnosed in the United States in 2009–2013. Neuro Oncol. 2016;18(suppl_5):v1–75. 28475809 10.1093/neuonc/now207 PMC8483569

[2] Buerki RA Horbinski CM Kruser T. An overview of meningiomas. Future Oncol. 2018;14:2161–77. 30084265 10.2217/fon-2018-0006 PMC6123887

[3] Lemee JM Corniola MV Da Broi M. Early postoperative complications in meningioma: predictive factors and impact on outcome. World Neurosurg. 2019;128:e851–8. 31082552 10.1016/j.wneu.2019.05.010

[4] Sughrue ME Rutkowski MJ Shangari G. Risk factors for the development of serious medical complications after resection of meningiomas. Clinical article. J Neurosurg. 2011;114:697–704. 20653395 10.3171/2010.6.JNS091974

[5] Zhao X Zhao D Wu Y. Meningioma in the elderly: characteristics, prognostic factors, and surgical strategy. J Clin Neurosci. 2018;56:143–9. 29958757 10.1016/j.jocn.2018.06.011

[6] Ko Y Park JH Yang MH. The significance of blood pressure variability for the development of hemorrhagic transformation in acute ischemic stroke. Stroke. 2010;41:2512–8. 20947842 10.1161/STROKEAHA.110.595561

[7] Chaodian L. Discussion of the causes and treatment of rebleeding after craniotomy for hypertensive cerebral hemorrhage. Chin Pharmacoeconomics. 2012;3:200–1.

[8] Rothwell PM Howard SC Dolan E. Prognostic significance of visit-to-visit variability, maximum systolic blood pressure, and episodic hypertension. Lancet. 2010;375:895–905. 20226988 10.1016/S0140-6736(10)60308-X

[9] Rothwell PM. Does blood pressure variability modulate cardiovascular risk? Curr Hypertens Rep. 2011;13:177–86. 21465141 10.1007/s11906-011-0201-3

[10] Liu W Qdaisat A Yeung J. The association between common clinical characteristics and postoperative morbidity and overall survival in patients with glioblastoma. Oncologist. 2019;24:529–36. 30049883 10.1634/theoncologist.2018-0056 PMC6459250

[11] Radinovic K Markovic Denic L Milan Z. Impact of intraoperative blood pressure, blood pressure fluctuation, and pulse pressure on postoperative delirium in elderly patients with hip fracture: a prospective cohort study. Injury. 2019;50:1558–64. 31279476 10.1016/j.injury.2019.06.026

[12] Bohm M Schumacher H Leong D. Systolic blood pressure variation and mean heart rate is associated with cognitive dysfunction in patients with high cardiovascular risk. Hypertension. 2015;65:651–61. 25583157 10.1161/HYPERTENSIONAHA.114.04568

[13] Gaujoux S Bonnet S Lentschener C. Preoperative risk factors of hemodynamic instability during laparoscopic adrenalectomy for pheochromocytoma. Surg Endosc. 2016;30:2984–93. 26684206 10.1007/s00464-015-4587-x

[14] Leoncini G Viazzi F Storace G. Blood pressure variability and multiple organ damage in primary hypertension. J Hum Hypertens. 2013;27:663–70. 23739158 10.1038/jhh.2013.45

[15] Whittle IR Smith C Navoo P. Meningiomas. Lancet. 2004;363:1535–43. 15135603 10.1016/S0140-6736(04)16153-9

[16] Peduzzi P Concato J Feinstein AR. Importance of events per independent variable in proportional hazards regression analysis. II. Accuracy and precision of regression estimates. J Clin Epidemiol. 1995;48:1503–10. 8543964 10.1016/0895-4356(95)00048-8

[17] Isobe N Ikawa F Tominaga A. Factors related to frailty associated with clinical deterioration after meningioma surgery in the elderly. World Neurosurg. 2018;119:e167–73. 30031190 10.1016/j.wneu.2018.07.080

[18] Dindo D Demartines N Clavien PA. Classification of surgical complications: a new proposal with evaluation in a cohort of 6336 patients and results of a survey. Ann Surg. 2004;240:205–13. 15273542 10.1097/01.sla.0000133083.54934.ae PMC1360123

[19] Katayama H Kurokawa Y Nakamura K. Extended Clavien-Dindo classification of surgical complications: Japan Clinical Oncology Group postoperative complications criteria. Surg Today. 2016;46:668–85. 26289837 10.1007/s00595-015-1236-x PMC4848327

[20] Woodfield JC Jamil W Sagar PM. Incidence and significance of postoperative complications occurring between discharge and 30 days: a prospective cohort study. J Surg Res. 2016;206:77–82. 27916378 10.1016/j.jss.2016.06.073

[21] Hanley C Ladha KS Clarke HA METS Study Investigators. Association of postoperative complications with persistent post-surgical pain: a multicentre prospective cohort study. Br J Anaesth. 2022;128:311–20. 34872718 10.1016/j.bja.2021.10.027

[22] Lu Z Zheng H Chen Z. Effect of etomidate vs propofol for total intravenous anesthesia on major postoperative complications in older patients: a randomized clinical trial. JAMA Surg. 2022;157:888–95. 35947398 10.1001/jamasurg.2022.3338 PMC9366659

[23] Hirsch J DePalma G Tsai TT. Impact of intraoperative hypotension and blood pressure fluctuations on early postoperative delirium after non-cardiac surgery. Br J Anaesth. 2015;115:418–26. 25616677 10.1093/bja/aeu458 PMC4533731

[24] Obisesan TO Obisesan OA Martins S. High blood pressure, hypertension, and high pulse pressure are associated with poorer cognitive function in persons aged 60 and older: the Third National Health and Nutrition Examination Survey. J Am Geriatr Soc. 2008;56:501–9. 18179496 10.1111/j.1532-5415.2007.01592.x PMC2614341

[25] Monk TG Bronsert MR Henderson WG. Association between intraoperative hypotension and hypertension and 30-day postoperative mortality in noncardiac surgery. Anesthesiology. 2015;123:307–19. 26083768 10.1097/ALN.0000000000000756

[26] Arima H Anderson CS Wang JG Intensive Blood Pressure Reduction in Acute Cerebral Haemorrhage Trial Investigators. Lower treatment blood pressure is associated with greatest reduction in hematoma growth after acute intracerebral hemorrhage. Hypertension. 2010;56:852–8. 20823381 10.1161/HYPERTENSIONAHA.110.154328

[27] Bangalore S Schwamm LH Smith EE Get With The Guidelines-Stroke Steering Committee and Investigators. Relation of admission blood pressure to in-hospital and 90-day outcomes in patients presenting with transient ischemic attack. Am J Cardiol. 2019;123:1083–95. 30685057 10.1016/j.amjcard.2018.12.037

[28] McCarthy DJ Ayodele M Luther E. Prolonged heightened blood pressure following mechanical thrombectomy for acute stroke is associated with worse outcomes. Neurocrit Care. 2020;32:198–205. 31385182 10.1007/s12028-019-00803-7

[29] Zhu KB Ye XZ Chen L. [Incidence and risk factors of delirium in patients post permanent pacemaker implantation]. Zhonghua Xin Xue Guan Bing Za Zhi. 2016;44:338–41. 27112613 10.3760/cma.j.issn.0253-3758.2016.04.012

[30] Hori D Brown C Ono M. Arterial pressure above the upper cerebral autoregulation limit during cardiopulmonary bypass is associated with postoperative delirium. Br J Anaesth. 2014;113:1009–17. 25256545 10.1093/bja/aeu319 PMC4235573

[31] Ono M Brady K Easley RB. Duration and magnitude of blood pressure below cerebral autoregulation threshold during cardiopulmonary bypass is associated with major morbidity and operative mortality. J Thorac Cardiovasc Surg. 2014;147:483–9. 24075467 10.1016/j.jtcvs.2013.07.069 PMC3865134

[32] Salmasi V Maheshwari K Yang D. Relationship between intraoperative hypotension, defined by either reduction from baseline or absolute thresholds, and acute kidney and myocardial injury after noncardiac surgery: a retrospective cohort analysis. Anesthesiology. 2017;126:47–65. 27792044 10.1097/ALN.0000000000001432

[33] Haas CE LeBlanc JM. Acute postoperative hypertension: a review of therapeutic options. Am J Health Syst Pharm. 2004;61:1661–73; quiz 1674. quiz16745. 15540477

[34] Muntner P Shimbo D Tonelli M. The relationship between visit-to-visit variability in systolic blood pressure and all-cause mortality in the general population: findings from NHANES III, 1988 to 1994. Hypertension. 2011;57:160–6. 21200000 10.1161/HYPERTENSIONAHA.110.162255

[35] Karhade AV Fandino L Gupta S. Impact of operative length on post-operative complications in meningioma surgery: a NSQIP analysis. J Neurooncol. 2017;131:59–67. 27864707 10.1007/s11060-016-2262-2

[36] Ekaireb RI Edwards CS Ali MS. Meningioma surgical outcomes and complications in patients aged 75 years and older. J Clin Neurosci. 2021;88:88–94. 33992210 10.1016/j.jocn.2021.03.032

[37] Hadanny A Tzubery S Hadelsberg U. The outcome of intracranial meningioma surgery in octogenarians: matched cohort study. World Neurosurg. 2020;144:e582–8. 32916350 10.1016/j.wneu.2020.09.001

