# Supplementary material for: Effect of systolic blood pressure fluctuations during resuscitation on postoperative complications following meningioma surgery: A retrospective observation study
Source: Medicine (Baltimore). 2022 Dec 9;101(49):e32259. doi: 10.1097/MD.0000000000032259 (PMC9750671; doi:10.1097/MD.0000000000032259)
Supplement: Supplementary file 1 [file medi-101-e32259-s001.pdf]

Table S1. Baseline characteristics of patients in the entire cohort and OR for POCs.

**Variables**	Entire cohort (n=578)	CDC grade < 2 (n=417)	CDC grade ≥ 2 (n=161)	OR for POCs (95% CI)	*P* value
Age, yr, mean (SD)	55.2 (0.5)	54.6 (0.5)	56.7 (0.9)	1.11 (1.00-1.03)	**0.045**
Female, sex, n (%)	445 (77.0)	324 (77.7)	121 (75.2)	0.87 (0.57-1.33)	0.515
BMI, kg/m^2, mean (SD)	24.3 (0.1)	24.3 (0.2)	24.3 (0.2)	1.00 (0.94-1.06)	0.867
Comorbidities, n (%)					
Hypertension	181 (31.3)	119 (28.5)	62 (38.5)	1.57 (1.07-2.31)	**0.021**
Diabetes mellitus	63 (10.9)	43 (10.3)	20 (12.4)	1.31 (0.77-2.23)	0.313
Neurological diseases	88 (15.2)	52 (12.5)	36 (22.4)	2.06 (1.29-3.31)	**0.003**
Cardiovascular disease	24 (4.2)	15 (3.6)	9 (5.6)	1.58 (0.68-3.69)	0.288
Underlying Diseases	319 (55.2)	213 (51.1)	106 (65.8)	1.85 (1.27-2.69)	**0.001**
Neurosurgical history, n (%)	51 (8.8)	32 (7.7)	19 (11.8)	1.51 (0.82-2.78)	0.184
Symptom duration, n (%)					
≤ 6 months	419 (72.5)	303 (72.7)	116 (72.0)	1.00 (reference)	
6 mouths-1 year	72 (12.5)	51 (12.2)	21 (13.0)	1.08 (0.62-1.87)	0.796
≥ 1 year	86 (14.9)	62 (14.9)	24 (15.0)	1.01 (0.60-1.70)	0.967
WHO classification, n (%)					
I	518 (89.6)	379 (90.9)	139 (86.3)	1.00 (reference)	
II	50 (8.7)	33 (7.9)	17 (10.6)	1.41 (0.76-2.60)	0.280

III	9 (1.6)	4 (0.1)	5 (3.1)	3.41 (0.90-12.88)	0.071
Maximum tumor diameter, mm, M (IQR) [a]	31 (23.3)	30.0 (20.0)	38.0 (23.8)	1.03 (1.01-1.04)	**<0.001**
Recurrent tumors, n (%)	49 (8.5)	32 (7.7)	17 (10.6)	1.42 (0.76-2.63)	0.270
Multiple tumors, n (%)	22 (3.8)	10 (2.4)	12 (7.5)	3.27 (1.38-7.73)	**0.007**
Preoperative anemia, n (%)	37 (6.4)	26 (6.2)	11 (6.8)	1.10 (0.53-2.29)	0.793
Hypoalbuminemia, n (%)	60 (10.4)	39 (9.4)	21 (13.0)	1.45 (0.83-2.59)	0.194

Data were presented as frequency (prevalence in %) or means (SD), or medians (IQR). Statistical analyses were performed using univariable logistic regression with no POCs as the reference group. Results are reported as OR for logistic regression analyses with 95% CIs. A P value of < .05 was statistically significant.

Abbreviations: POCs, postoperative complications; CDC, Clavien-Dindo Classification; BMI, Body mass index; WHO, World Health Organization; OR, odds ratio; 95% CI, 95% confidence interval；M, Median; IQR, inter quartile range; SD, Standard deviations.

[a] missing data.

Table S2. Perioperative characteristics of patients in the entire cohort and OR for POCs.

**Variables**	Entire cohort (n=578)	CDC grade < 2 (n=417)	CDC grade ≥ 2 (n=161)	OR for POCs (95% CI)	*P* value
ASA classification, n (%)					
II	528 (91.3)	387 (92.8)	141 (87.6)	1.00 (reference)	
III	48 (8.3)	28 (6.7)	20 (12.4)	1.92 (1.07-3.59)	**0.029**
IV	1 (0.2)	1 (0.2)	0 (0.0)	-	-
Start time of surgery, n (%)					
Before 12pm	393 (68.0)	272 (65.2)	121 (75.2)	1.00 (reference)	
After 12pm	185 (32.0)	145 (34.8)	40 (24.8)	0.62 (0.41-0.94)	**0.022**
Anesthesia time, min, mean (SD)	267.9 (4.2)	256.4 (4.6)	302.6 (8.75)	1.01 (1.00-1.01)	**<0.001**
Operation time, min, mean (SD)	236.9 (4.1)	226.1 (4.5)	269.5 (8.47)	1.01 (1.00-1.01)	**<0.001**
Intraoperative hypertension, n (%)	141 (24.4)	94 (22.5)	47 (29.2)	1.42 (0.94-2.32)	0.096
Anesthetic drugs, M (IQR)					
Propofol	1360 (630)	1310 (620)	1860 (960)	1.00 (1.00-1.00)	**<0.001**
Sufentanil	40.0 (15)	40.0 (10)	40.0 (15)	1.01 (0.99-1.02)	0.479
Rifentanil	1 (0.0)	1 (0.0)	1 (1.0)	3.54 (2.23-5.63)	**<0.001**
Atracurium	30 (10)	30 (10)	35 (20)	1.04 (1.02-1.05)	**<0.001**
Dexmedetomidine, n (%)	269 (46.5)	190 (45.6)	79 (49.1)	1.15 (0.80-1.66)	0.449

NSAIDS, n (%)	552 (95.5)	382 (91.6)	140 (87.0)	0.59 (0.33-1.06)	0.076
Total volume, ml, M (IQR)	1638 (500)	1500 (500)	2000 (1000)	1.00 (1.00-1.00)	**<0.001**
Crystal volume, ml, M (IQR)	1000 (363)	1000 (300)	1000 (500)	1.00 (1.00-1.00)	**<0.001**
Colloidal volume, ml, M (IQR)	500 (500)	500 (500)	800 (500)	1.00 (1.00-1.00)	**<0.001**
Intraoperative mannitol，n (%)	44 (7.6)	26 (6.2)	18 (11.2)	1.95 (1.05-3.73)	**0.036**
Total blood loss, ml, M (IQR)	300 (300)	300 (200)	400 (300)	1.00 (1.00-1.00)	**<0.001**
Blood transfusion, n (%)	80 (13.8)	47 (11.3)	33 (20.5)	2.03 (1.25-3.31)	**0.005**
Endotracheal unextubation, n (%)	58 (10.0)	26 (6.2)	32 (19.9)	4.79 (2.40-9.56)	**<0.001**
Resuscitation time, min, mean (SD)	98.2 (1.4)	96.0 (1.5)	104.7 (3.3)	1.00 (1.00-1.01)	0.128
Supplementary antihypertensive drugs, n (%)	15 (2.6)	9 (2.2)	6 (3.7)	1.76 (0.61-5.01)	0.294
Supplementary propofol, n (%)	123 (21.3)	86 (20.6)	37 (23.0)	1.15 (0.74-1.78)	0.535
SBPV, mean (SD)	6.72 (0.1)	6.20 (0.2)	8.07 (0.3)	1.18 (1.12-1.25)	**<0.001**
DBPV, mean (SD)	7.15 (0.1)	7.09 (0.2)	7.30 (0.2)	1.03 (0.97-1.09)	0.418

Data were presented as frequency (prevalence in %) or means (SD), or medians (IQR). Statistical analyses were performed using univariable logistic regression with no POCs as the reference group. Results are reported as OR for logistic regression analyses with 95% CIs. A P value of < .05 was statistically significant.

Abbreviations: POCs, postoperative complications; CDC, Clavien-Dindo Classification; ASA, American Society of Anesthesiologists Physical Status Classification; NSAID, nonsteroidal anti-inflammatory drug; SBPV, systolic blood pressure variability; DBPV, diastolic blood pressure

variability; OR, odds ratio; 95% CI, 95% confidence interval；M, Median; IQR, inter quartile range; SD, Standard deviations.

Table S3. Postoperative events of patients in the entire cohort.

	n=578
**Major interventions, n(%)**	
Secondary surgery	14 (2.4)
Postoperative blood transfusion	16 (2.8)
Tracheotomy and ventilator-assisted breathing	16 (2.8)
Parenteral nutrition	4 (0.7)
Overall in-hospital POCs, n(%)	308 (53.3)
CDC grade, M (IQR)	1.0 (2)
CDC grade ≥ 2, n(%)	161 (27.9)
**Laboratory metrics, n(%)**	
Low sodium and potassium	72 (12.5)
Low hemoglobin	178 (30.8)
High leukocytes	492 (85.1)
**Specific POCs, n(%)**	
Persistent fever	98 (17.0)
Intracranial infections	42 (7.3)
Pulmonary infections	42 (7.3)
Cranial nerve damage	40 (6.9)
Seizures	14 (2.4)
Cerebrospinal fluid leakage	10 (1.7)

Consciousness Disorders	34 (5.9)
Incision infections/splitting	12 (2.1)
Speech Disorders	16 (2.8)
Acute cerebral infarction	23 (4.0)
Epidural hematoma	26 (4.5)
Intracranial hemorrhage/hematoma	32 (5.5)
Severe cerebral edema	24 (4.2)
Subscalp fluid accumulation	67 (11.6)
NICU LOS, M (IQR)	1 (0)
PLOS, M (IQR)	10 (6)
Total LOS, M (IQR)	16 (8)
30 Day readmission, n(%)	11 (1.9)
90 Day readmission, n(%)	20 (3.5)
90-day length of hospitalization, M (IQR)	0 (0)
Total hospitalization costs, yuan, mean (SD)	81120 (1116.3)

Data were presented as frequency (prevalence in %) or means (SD), or medians (IQR). **Abbreviations:** POCs, postoperative complications; CDC, Clavien-Dindo Classification; NICU, Neurosurgical Intensive Care Unit; LOS, length of stay; PLOS, postoperative length of stay; M, Median; IQR, inter quartile range; SD, Standard deviations.

Table S4. Specific POCs and CDC grades of patients in the entire cohort.

POCs	CDC grade	Frequency
Electrolyte disturbance	I	2
Persistent fever	I	105
Hyperpotassemia	I	2
Acidosis	I	1
Urinary retention	I	4
Abdominal effusion	I	1
Abdominal distension	I	1
Hypokalemia	I	36
Hyponatremia	I	32
Consciousness Disorders	I	34
Pleural effusion	I	15
Pulmonary edema	I	1
Hypoproteinemia	II	78
Hypoalbuminemia	II	2
Acute thrombocytopenia	II	1
Throat infections	II	2
Incision infections	II	12
Pulmonary infections	II	42
Stress ulcer complicated with hemorrhage	II	1
Intracranial infections	II	42

Venous thrombosis in lower extremity	II	2
Cranial nerve damage	II	20
Seizures	II	14
Speech Disorders	II	16
Cerebrospinal fluid leakage	II	10
Bacteremia	II	3
Subscalp fluid accumulation	II	45
Arrhythmia	II	1
Gastrointestinal dysfunction	II	6
Wound splitting	II	12
Acute kidney injury	II	1
Atrial fibrillation	II	4
Chronic bronchitis	II	2
Upper respiratory tract infection	II	5
Urinary tract infection	II	3
Postoperative blood transfusion	II	16
Parenteral nutrition	II	4
Stress ulcer complicated with hemorrhage	III a	1
Secondary surgery (local anesthesia)	III a	2
Tracheotomy (local anesthesia)	III a	16
Secondary surgery (general anesthesia)	III b	14
Acute cerebral infarction	IV a	21

Severe epidural hematoma	IV a	12
Severe intracranial hemorrhage/hematoma	IV a	14
Respiratory failure	IV a	5
Heart failure	IV a	2
Acute renal insufficiency	IV a	3
Acute cardiac insufficiency	IV a	3
Brain herniation	IV a	2
Transferred to NICU	IV a	1
Toxic shock induced by infection	IV b	2
Multiple organ failure	IV b	3
Death	V	2

Data of specific POCs were presented as frequency (prevalence in %).

Abbreviations: POCs, postoperative complications; CDC, Clavien-Dindo Classification; NICU, Neurosurgical Intensive Care Unit.
