# Supplementary material for: Effect of systolic blood pressure fluctuations during resuscitation on postoperative complications following meningioma surgery: A retrospective observation study
Source: Medicine (Baltimore). 2022 Dec 9;101(49):e32259. doi: 10.1097/MD.0000000000032259 (PMC9750671; doi:10.1097/MD.0000000000032259)
Supplement: Supplementary file 2 [file medi-101-e32259-s002.pdf]

**Table S5.** Univariable and multivariable analyses of the association between SBPV and POCs, as a sensitivity analysis in the entire cohort.

| SBPV quartiles/continuous | Number of events | Crude OR (95% CI) | Crude *P*-value | Adjusted OR (95% CI) | Adjusted *P*-value |
|---|---|---|---|---|---|
| Q1 | 144 | Reference | NA | Reference | NA |
| Q2 | 145 | 1.53 (0.83-2.82) | 0.175 | 1.69 (0.89-3.24) | 0.111 |
| Q3 | 144 | 2.41 (1.34-4.33) | 0.003 | 2.36 (1.26-4.39) | 0.007 |
| Q4 | 144 | 5.24 (2.97-9.24) | < 0.001 | 4.65 (2.53-8.57) | < 0.001 |
| ln | 578 | 3.95 (2.56-6.09) | < 0.001 | 3.39 (2.16-5.31) | < 0.001 |

The systolic blood pressure variability values were transformed as a natural log value and quartiles. Statistical analyses were performed using univariable and multivariable logistic regression with no POCs as the reference group. Results are reported as crude OR or adjusted OR for logistic regression analyses with 95% CIs. A P value of < .05 was statistically significant.

**Abbreviations:** SBPV, systolic blood pressure variability; POCs, postoperative complications; OR,odds ratio; 95% CI, 95% confidence interval； NA, not available.
