# Supplementary material for: Effect of systolic blood pressure fluctuations during resuscitation on postoperative complications following meningioma surgery: A retrospective observation study
Source: Medicine (Baltimore). 2022 Dec 9;101(49):e32259. doi: 10.1097/MD.0000000000032259 (PMC9750671; doi:10.1097/MD.0000000000032259)
Supplement: Supplementary file 3 [file medi-101-e32259-s003.pdf]

Table S6. Univariable and multivariable linear regression analysis associated with PLOS.

Risk factors	Univariate analysis		Multivariate analysis	
	β	*P* value	*β* (95% CI)	*P* value
Age	0.07	**0.022**	-	-
Hypertension	2.41	**0.002**	2.02 (0.28-3.75)	0.023
Neurological diseases	2.57	**0.009**	-	-
Gastrointestinal disease	3.71	**0.004**	2.79 (0.47-5.11)	0.018
Comorbidities	2.63	**< 0.001**	-	-
With symptoms	2.03	0.058	-	-
Symptom duration	1.25	**0.010**	-	-
WHO classification	2.15	**0.026**	-	-
Maximum tumor diameter [a]	0.12	**< 0.001**	-	-
Recurrent tumors	2.38	0.062	-	-
ASA classification	2.91	**0.018**	-	-
Surgical position	0.80	0.086	-	-
Operation time ≥ 3h	0.03	**< 0.001**	2.42 (0.58-4.27)	0.010
Anesthetic drugs				
Propofol	0.01	**< 0.001**	-	-
Sufentanil	0.08	**0.014**	-	-
Rifentanil	6.93	**< 0.001**	-	-
Atracurium	0.17	**< 0.001**	-	-

Total liquid	0.01	**< 0.001**	1.62 (0.01-3.25)	0.050
Total blood loss	0.01	**< 0.001**	-	-
Blood transfusion	2.82	**0.006**	-	-
Endotracheal unextubation	5.73	**< 0.001**	-	-
SBPV	0.87	**< 0.001**	1.86 (0.58-3.13)	0.004
DBPV	0.32	**0.008**	-	-
NICU LOS	1.63	**< 0.001**	2.59 (1.03-4.15)	0.001
CDC grade ≥ 2	7.70	**< 0.001**	4.72 (3.28-6.16)	< 0.001
A sensitivity analysis of SBPV				
**Model 1** [b]			1.94 (0.63-3.24)	0.004
**Model 2** [c]			1.74 (0.49-2.99)	0.006

Statistical analyses were performed using univariable and multivariable linear regression. Results are reported as adjusted β valve for linear regression analyses with 95% CIs. A P value of < .05 was statistically significant.

Abbreviations: PLOS, postoperative length of stay; WHO, World Health Organization; ASA, American Society of Anesthesiologists Physical Status Classification; SBPV, systolic blood pressure variability; DBPV, diastolic blood pressure variability; NICU, Neurosurgical Intensive Care Unit; LOS, length of stay; CDC, Clavien-Dindo Classification; 95% CI, 95% confidence interval.

[a] missing data.
