# Supplementary material for: Effect of systolic blood pressure fluctuations during resuscitation on postoperative complications following meningioma surgery: A retrospective observation study
Source: Medicine (Baltimore). 2022 Dec 9;101(49):e32259. doi: 10.1097/MD.0000000000032259 (PMC9750671; doi:10.1097/MD.0000000000032259)
Supplement: Supplementary file 4 [file medi-101-e32259-s004.pdf]

Table S7 Baseline and perioperative covariates before and after matching

Variables	Level	Before Matching			After Matching		
		Non-POC	POC	ASD△	Non-POC	POC	ASD△
n		417	161		205	137	
Age (mean (SD))		54.57 (10.84)	56.67 (12.19)	0.172	56.30 (10.35)	56.46 (11.77)	-0.025
HBP (%)	No	303 (72.7)	101 (62.7)	-0.205	132 (64.4)	84 (61.3)	-0.015
	Yes	114 (27.3)	60 (37.3)	0.205	73 (35.6)	53 (38.7)	0.015
Neurological.diseases (%)	No	366 (87.8)	125 (77.6)	-0.243	172 (83.9)	114 (83.2)	0.018
	Yes	51 (12.2)	36 (22.4)	0.243	33 (16.1)	23 (16.8)	-0.018
Comorbidities (%)	No	204 (48.9)	55 (34.2)	-0.311	79 (38.5)	49 (35.8)	0.015
	Yes	213 (51.1)	106 (65.8)	0.311	126 (61.5)	88 (64.2)	-0.015
brain.surgery (%)	No	385 (92.3)	143 (88.8)	-0.111	187 (91.2)	125 (91.2)	-0.023
	Yes	32 (7.7)	18 (11.2)	0.111	18 (8.8)	12 (8.8)	0.023
Diameter (mean (SD))		31.41 (14.74)	37.81 (15.95)	0.401	33.89 (14.68)	35.99 (14.12)	0.069
Recurrent.tumor (%)	No	385 (92.3)	144 (89.4)	-0.094	188 (91.7)	126 (92.0)	-0.012
	Yes	32 (7.7)	17 (10.6)	0.094	17 (8.3)	11 (8.0)	0.012
Multiple.tumor (%)	No	407 (97.6)	149 (92.5)	-0.192	197 (96.1)	130 (94.9)	0.014
	Yes	10 (2.4)	12 (7.5)	0.192	8 (3.9)	7 (5.1)	-0.014

Variables	Level	Before Matching			After Matching		
		Non-POC	POC	ASD$^{\Delta}$	Non-POC	POC	ASD$^{\Delta}$
WHO (%)	1	380 (91.1)	139 (86.3)	-0.140	183 (89.3)	121 (88.3)	0.000
	2	33 (7.9)	17 (10.6)	0.086	18 (8.8)	13 (9.5)	-0.000
	3	4 (1.0)	5 (3.1)	0.124	4 (2.0)	3 (2.2)	0.000
ASA (%)	2	388 (93.0)	141 (87.6)	-0.166	185 (90.2)	122 (89.1)	-0.011
	3	28 (6.7)	20 (12.4)	0.173	20 (9.8)	15 (10.9)	0.011
	4	1 (0.2)	0 (0.0)	-0.058	0 (0.0)	0 (0.0)	0.000
Start time of surgery (%)	AM	272 (65.2)	121 (75.2)	0.230	156 (76.1)	101 (73.7)	-0.093
	PM	145 (34.8)	40 (24.8)	-0.230	49 (23.9)	36 (26.3)	0.093
Operation.time (mean (SD))		230.35 (91.45)	303.81 (140.98)	0.521	259.49 (102.18)	272.50 (102.58)	0.003
Intra.HBP (%)	No	323 (77.5)	114 (70.8)	-0.146	154 (75.1)	100 (73.0)	-0.032
	Yes	94 (22.5)	47 (29.2)	0.146	51 (24.9)	37 (27.0)	0.032
Total.volume (mean (SD))		1741.45 (746.89)	2263.09 (1255.71)	0.415	1925.46 (879.00)	2012.66 (792.19)	-0.015
Blood.loss (mean (SD))		355.35 (239.64)	526.83 (525.94)	0.326	413.71 (282.42)	418.76 (240.82)	-0.041
Blood Transfusion (%)	No	370 (88.7)	128 (79.5)	-0.229	181 (88.3)	118 (86.1)	0.009
	Yes	47 (11.3)	33 (20.5)	0.229	24 (11.7)	19 (13.9)	-0.009

Variables	Level	Before Matching			After Matching		
		Non-POC	POC	ASD[△]	Non-POC	POC	ASD[△]
Endotracheal Unextubation (%)	No	403 (96.6)	138 (85.7)	-0.312	194 (94.6)	124 (90.5)	-0.063
	Yes	14 (3.4)	23 (14.3)	0.312	11 (5.4)	13 (9.5)	0.063

[△] Absolute Standardized Difference (ASD): absolute difference in means or proportions divided by the pooled SD; ASD values of 0.2, 0.5, and 0.8 represent small, median, and large differences.

Abbreviations: PSM, propensity score matching; POCs, postoperative complications; WHO, World Health Organization; ASA, American Society of Anesthesiologists Physical Status Classification; SBPV, systolic blood pressure variability；ASD, Absolute Standardized Differenc; SD, Standard deviations.
