# Supplementary material for: Effect of systolic blood pressure fluctuations during resuscitation on postoperative complications following meningioma surgery: A retrospective observation study
Source: Medicine (Baltimore). 2022 Dec 9;101(49):e32259. doi: 10.1097/MD.0000000000032259 (PMC9750671; doi:10.1097/MD.0000000000032259)

Figure S1. Subgroup analysis of the association between SBPV and POCs.

Abbreviations: SBPV, systolic blood pressure variability; POCs, postoperative complications; WHO, World Health Organization.

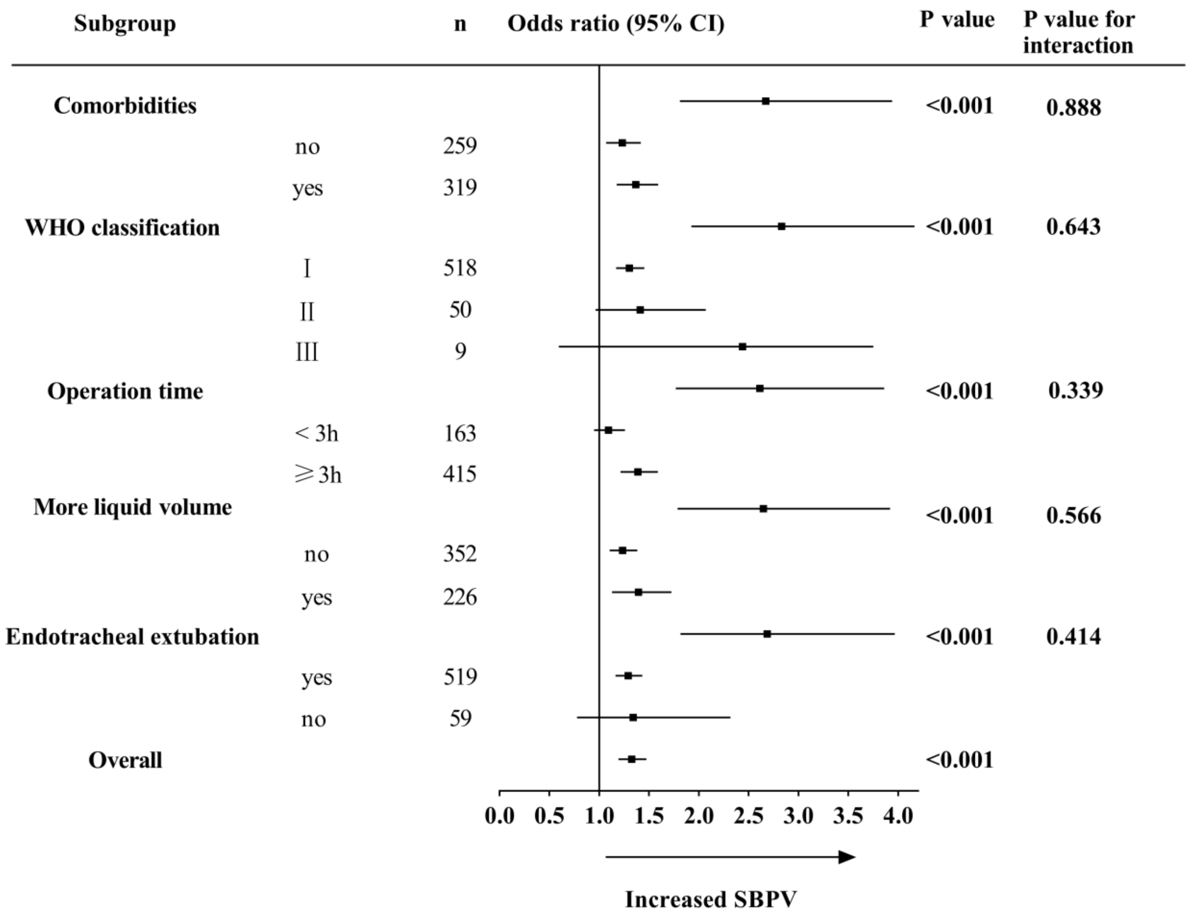

Supplement: Supplementary file 5 [file medi-101-e32259-s005.pdf]
