# Supplementary material for: Effect of systolic blood pressure fluctuations during resuscitation on postoperative complications following meningioma surgery: A retrospective observation study
Source: Medicine (Baltimore). 2022 Dec 9;101(49):e32259. doi: 10.1097/MD.0000000000032259 (PMC9750671; doi:10.1097/MD.0000000000032259)

Figure S2. The matching effect of each covariate before and after propensity score matching.

Abbreviations: SBPV, systolic blood pressure variability; ASA, American Society of Anesthesiologists Physical Status Classification; WHO, World Health Organization;

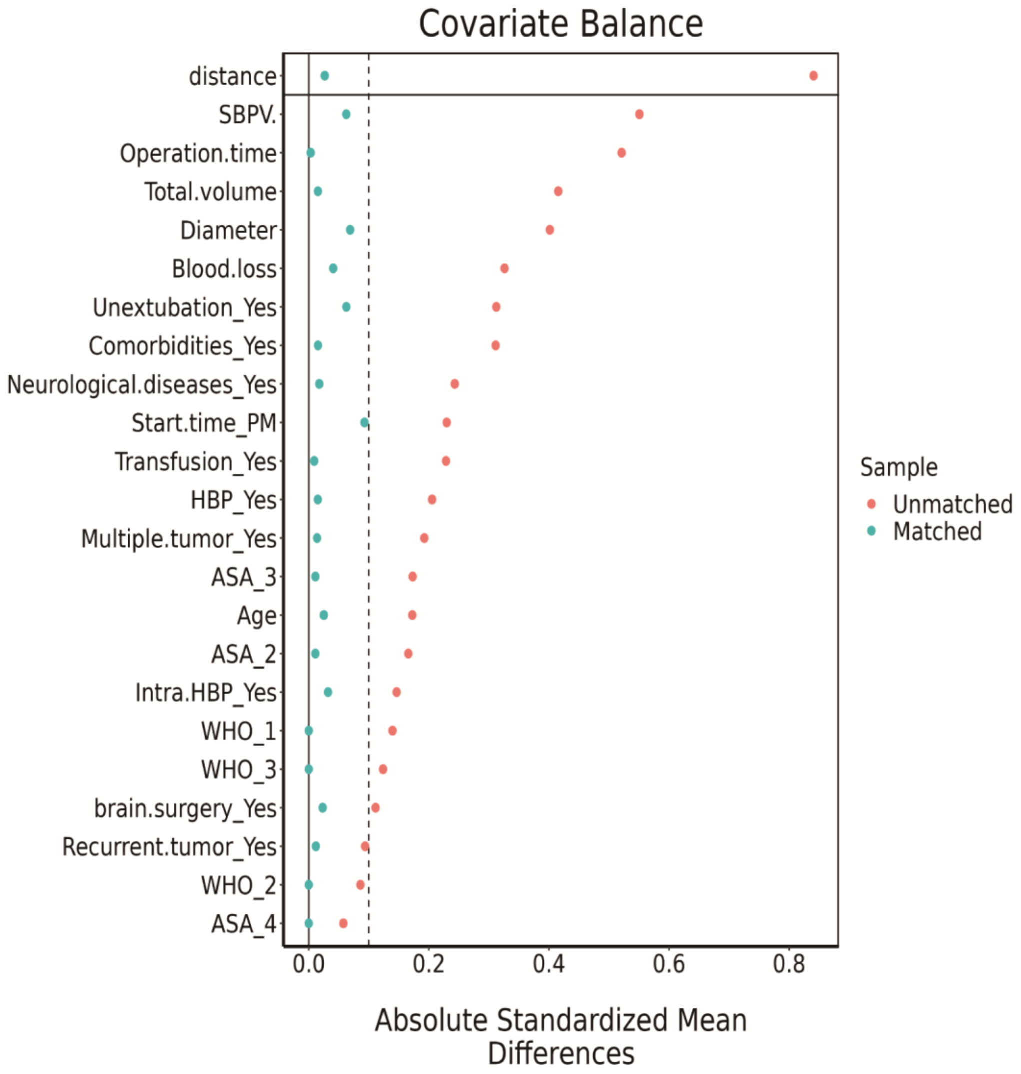

Supplement: Supplementary file 6 [file medi-101-e32259-s006.pdf]
